# The potential role of the educational system in addressing the effect of inadequate knowledge of mosquitoes on use of insecticide-treated nets in Ghana

**DOI:** 10.1186/1475-2875-9-256

**Published:** 2010-09-15

**Authors:** Andreas A Kudom, Ben A Mensah

**Affiliations:** 1 Department of Entomology and Wildlife, School of Biological Sciences, University of Cape Coast, Ghana

## Abstract

**Background:**

Since 2001, there has been a tremendous increase in number of households protected by ITN and IRS in Ghana. However, there has not been evidence of a reduction in malaria cases as expected and reported deaths have rather increased since 2007. As a result, this study was undertaken to get a better understanding of perceptions of malaria, knowledge on mosquitoes and the value attached to ITNs among secondary and tertiary students in Cape Coast.

**Methods:**

Structured questionnaires were administered randomly to gather data on demographic characteristics of students, knowledge of mosquitoes and ITNs and attitude towards the use of ITN in seven public high schools and four tertiary institutions in Cape Coast metropolis. In addition, curriculums of science courses common to all students from junior high school to the university were carefully examined.

**Results:**

A total of 492 students took part in this study and more than 90% of them had high knowledge of malaria transmission and ITN, but little knowledge of mosquito life history. Only 1% in secondary and 2.1% in tertiary institutions had seen or knew about all the development stages of mosquitoes. In high school and tertiary institutions, 24.2% and 10.8% of respondents, respectively, were able to mention other genera of mosquitoes, apart from *Anopheles*. Though 93.9% in senior high school and 86.7% in the tertiary institutions knew that ITNs are either used to protect oneself from mosquito bites or to prevent malaria, 32.7% of the respondents in secondary and 21.9% in tertiary institutions who owned ITN did not use them.

**Conclusions:**

The study reveals that respondents did not have adequate knowledge on the biology and behaviour of mosquitoes. This appears to weaken their knowledge of the link between the use of ITN and malaria control; the effect of this is that a significant number owned ITNs but did not use them. The implication is that if people will really accept and use ITN or other mosquito control interventions, then just creating awareness of those interventions is not enough but people should also be educated on the life history of mosquitoes and on the mechanism of the control strategies. This can be effectively done through the formal education system.

## Background

Current policy options for malaria control include prompt and effective disease treatment and disease prevention through use of insecticide-treated net (ITN) [1]. A number of studies have demonstrated that the use of ITN is effective in reducing malaria-related morbidity and mortality [2,3]. The use of ITNs on a large scale reduced clinical malaria episodes by 48% and saved 6% of 1,000 children below five years of age [4]. Although these trials have demonstrated that ITNs are an effective malaria control strategy, there have been many challenges to ITN distribution, acceptance and utilization especially when trying to implement large-scale ITN programmes [3]. Knowledge about the cause of malaria and about the existence of ITNs was low in many malaria-endemic communities [5]. High cost and lack of access were some of the reasons stated as to why ITNs were not used [6].

In Ghana, malaria accounts for at least 20% of child deaths, 40% of admissions of children to hospital and more than 50% of out-patient attendance [7]. The current strategy of the National Malaria Control Programme involves the use of ITN and indoor residual spraying of insecticide (IRS). Since 2001, there has been a tremendous increase in number of households protected by ITN and IRS. For instance, the operational coverage of the ITNs rose from 742,000 in 2002 to more than 1,477,000 in 2007. However, there has not been evidence of a reduction in malaria cases, and reported deaths have increased since 2007 [8].

Knowledge of people's perceptions of malaria and of the socio-economic implications of the disease is of considerable value when control programmes are being planned and implemented. Health education appears to be improving malaria-specific knowledge, which in turn is reportedly having some positive impact on ITN ownership at the household level [9]. The main achievement in this area has been the raising of awareness especially on the causation, transmission and symptoms of malaria [9,10], and improved knowledge is leading to some minimal behaviour change, both in use of anti-malarial drugs and in ownership of bed nets [9-12]. However, awareness and improvement in knowledge concerning the above-mentioned parameters have not yet been translated into actions that could reverse the upward trend in incidence of malaria in any significant way.

This study was undertaken to get a better understanding of people's perceptions of malaria, its cause and prevention and the value attached to ITNs. The main objectives were to relate awareness of malaria to the use of ITN among secondary and tertiary students in Cape Coast, Ghana, and to assess basic knowledge on life history of mosquitoes among the students and the effect of that knowledge on the use of ITN.

## Methods

The study was undertaken in Cape Coast Metropolis, the capital of Central Region of Ghana. It is situated 165 km west of Accra (capital of Ghana) on the Gulf of Guinea (5°06'N 1°15'W; 5.1°N 1.25°W). The Metropolis has a population of 118,106 (2000 census). The metropolis occupies approximately 122 square kilometres of land and experiences a temperature range of 21-36°C throughout the year, with a double maxima rainfall totalling between 750 mm and 1,000 mm. The major rainy season is between May and July and the minor rainy season between November and January. Cape Coast is a humid area with mean monthly relative humidity varying between 85% and 99%. Reported malaria cases in the metropolis rose from 61,077 in 2007 to 109,211 in 2009 [13]. There are 11 public high schools and five tertiary institutions in the metropolis, including teachers and nurses training colleges, a polytechnic and a University.

The research was carried out between December, 2009 and April 2010, in seven public high schools (HS) and four tertiary schools (TS) with well-organized hostel or boarding facilities which were managed by the school authorities. In each school, fifty structured questionnaires were administered randomly to gather data on demographic characteristics of students, knowledge of ITNs and attitude to their use. The students' hostels in each of the schools were physically inspected for the presence or absence of mosquito nets. Where present, nets were counted and their types noted. The states of the nets were also checked for torn parts and whether they were fixed properly. Insecticide-treated nets involved in this experiment were purchased by the students for use in school.

In addition, curriculums of science courses common to all students from junior high to the university were reviewed, to assess the contribution they could make to the education of students on malaria. Integrated Science for high school and Science for teacher training college were the subjects reviewed. Integrated science is a compulsory course for all high school students that introduce students to basic sciences.

The data from the research were processed using the Statistical Package for Social Science (SPSS, version 15). Parameters were compared between senior high schools and tertiary institutions using Chi square test at 95% confidence interval with StatCalc application software (version 6).

### Ethical consideration

Informed consent was sought and obtained from all study participants after a standard explanation of the study objectives had been clearly spelled out and confidentiality was assured. All protocols followed were in line with the ethics requirements of the University of Cape Coast, Ghana Education Service and Ghana Health Service.

## Results

### Demographic characteristics

A total of 492 students from seven high schools and four tertiary schools in the Cape Coast metropolis were involved in this study. In the high school, majority (94.2%) were in the age range of 14-20 while in tertiary institutions, the majority (91.2%) were between 20 and 30. Sex distribution showed almost 1:1 ratio in the high school and about 1:2 (male: female) in the tertiary institutions (Table 1). Out of the seven high schools, three had male students only, two had female students only and two had students of both sexes. Two of the tertiary schools were female institutions and the other two were mixed.

**Table 1:** Demographic characteristics of respondents from senior high schools and tertiary institutions in Cape Coast Metropolis.

Educational level		Age range (%)				Sex (%)		Program (%)	
				
	N	<14	14-20	20-30	>30	male	female	science	other
High school	297	0.3	94.2	5.5	0	55.6	44.4	30.4	69.6
Tertiary	195	0	6.7	91.2	2.1	34.5	65.5	68.6	31.4

### Knowledge of basic life history of mosquitoes

**Figure 1:**
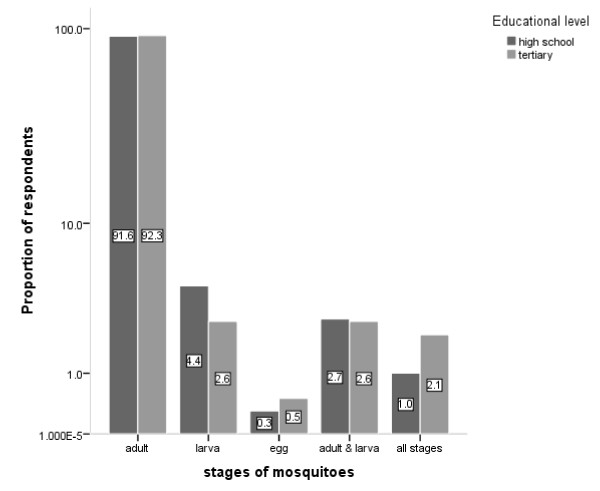
**Mosquito developmental stages ever seen by the respondents in senior high schools and tertiary institutions in Cape Coast Metropolis (insert: % proportion of respondents)**.

All the respondents (100%) in both high school and tertiary institutions had seen mosquitoes before but only 1% and 2.1%, respectively, had seen or knew about all the development stages of mosquitoes (Figure 1). Majority of students in high school (91.6%) and tertiary institution (92.3%) had only seen adult mosquitoes. Proportions of respondents who had seen either the adult stage or all the stages of development of mosquitoes were similar in high school and tertiary institutions (p = 0.953; p = 0.348 respectively). Again, similar proportions of respondents in high school and tertiary institutions knew that there are different types of mosquitoes (p = 0.916). However, only 24.2% of respondents in high school were able to name other genera of mosquitoes (such as *Culex *or *Aedes*) apart from *Anopheles*, which was significantly higher than 10.8% respondents in tertiary institutions (p = 0.001). Majority of respondents at both levels of education either did not know that there are other genera of mosquitoes apart from *Anopheles *or could not name or describe any genus of mosquitoes apart from *Anopheles *(Table 2).

**Table 2:** Knowledge of different types of mosquitoes among students in senior high schools and tertiary institutions in Cape Coast Metropolis.

Educational level	Different types of mosquitoes			Name or describe types of mosquitoes	
		
	Yes (%)	No (%)	No idea (%)	Able (%)	Unable (%)
High school	86.9	11.4	1.7	24.2	75.8
Tertiary	85.6	10.3	4.1	10.8	89.2

### Knowledge on malaria transmission

In the high school, 96.9% of the respondents dislike mosquitoes and 82.6% of these cited transmission of diseases as their reason. In addition, 96.7% mentioned malaria as one of the diseases transmitted by mosquitoes and 0.7% added elephantiasis. Furthermore, 88.4% were aware that mosquitoes transmit diseases through biting. Nevertheless, when respondents were asked what they knew about malaria, only 41.8% linked mosquitoes to the disease. There were similar responses from the tertiary institutions: 99% expressed dislike for mosquitoes, 94.4% said they transmit diseases, [97.4% out of these cited malaria as an example of diseases transmitted by mosquitoes] and 89.7% knew that this disease is transmitted through biting. But in a reverse question (knowledge on malaria), 50.5% linked mosquitoes to malaria; this response was not significantly different from the response obtained from students in senior high schools (p = 0.199) (Table 3).

**Table 3:** Knowledge on malaria and the role of mosquitoes in malaria transmission among students in senior high schools and tertiary institutions in Cape Coast Metropolis

Educational level	Why dislike mosquitoes		How mosquito transmit diseases		Knowledge on malaria	
			
	Diseases/bite (%)	Other (%)	Bite (%)	Other (%)	Mosquito linked (%)	Other (%)
High school	82.6	17.4	88.4	11.6	41.8	58.2
Tertiary	94.4	5.6	89.7	10.3	50.5	49.5

### Ownership and the use of ITN

**Figure 2:**
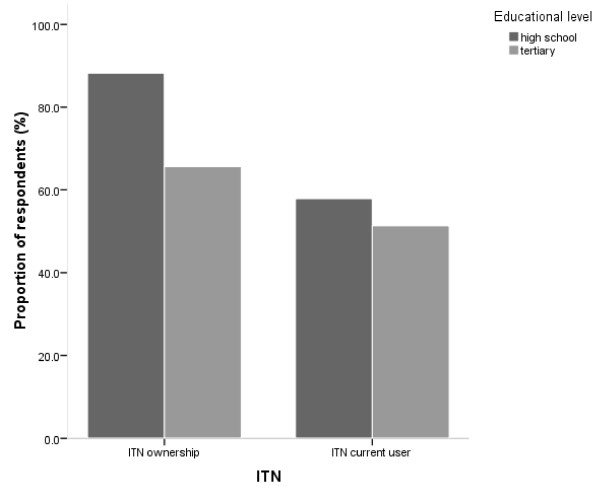
**Proportion of students in senior high schools or tertiary institutions in Cape Coast who owned or used ITNs**.

When the respondents were asked what they knew about ITN, 93.9% in the senior high school and 86.7% in the tertiary institutions said that it is either used to protect oneself from mosquito bites or to prevent malaria. Accordingly, 79.8% of the respondents in senior high schools and 86.9% in tertiary institutions mentioned ITN as an effective strategy for protection against mosquito bites. In the senior high schools, 98.6% have seen ITN and 92.9% have used it. One school had a policy that made the use of ITN compulsory, hence there was almost 100% ownership and use of ITN. However, 86.1% owned ITN in the remaining senior high schools but 32.7% of the respondents that owned ITN did not use them. There was a significant difference between ITN ownership and use (p = 0.0043) (Figure 2). There was also a significant difference between respondents who had used ITNs before and those who currently use them (p = 0.0003).

Similar results were obtained in tertiary institutions; there was no significant difference between the 99.0% of respondents who had seen ITN and the 83.1% who had used it before (p = 0.234). The proportion of respondents who owned ITN (65.6%) was, however, not different from those who used them (51.3%) (p = 0.14), though 21.9% of respondents that owned ITN did not use them. Furthermore, the difference between the respondents who once used ITN and those currently use them is significant (p = 0.0029). Similar proportions of respondents at senior high and tertiary level had seen (p = 0.979) and used (p = 0.407) ITN before or own (p = 0.447) and currently use (p = 0.064) ITN.

Reasons given by non-ITN-users included cost, heat, and discomfort when sleeping under it. In addition, some claimed mosquitoes bit them before they went to bed so there was no need to sleep under ITN.

In the tertiary institutions, 69.7% of students in nurses training colleges, 31.8% of students in the teacher training college and 20.3% of students in one hall of residence of the university used ITN. There was a significant difference between the actual use of ITN in the nurses training colleges and the teacher training college (p < 0.0001) or the university (p < 0.0001). All the nets were long-lasting insecticide nets, including Permanet^®^, Olyset^® ^and Interceptor^®^, which were of high quality and were fixed properly.

After the curriculum was reviewed, 'Integrated Science' was found to be a common science course to all students from junior high to senior high school. Science as a course was also common to all students in the teachers training college under this study. In the university, there was no science course which was common to all students.

## Discussion

A good understanding of people's perceptions of malaria and its cause, preventive action, and value attached to ITNs are important determinants of success in malaria control programs that promote ITN use. The results from this study have shown that students in the senior high schools and tertiary institutions in Cape Coast Metropolis have high knowledge of malaria transmission and ITN, but little knowledge of mosquito life history. High knowledge and awareness of malaria and ITN are consistent with the findings of a similar study among secondary school students in Zaria, Northern Nigeria [14].

Knowledge of the life history of the vector is as important as knowing that mosquitoes transmit malaria. Vector control aims to decrease contact between humans and vectors of human disease. Control of mosquitoes may prevent malaria as well as several other mosquito-borne diseases but mosquitoes cannot be effectively controlled without knowledge of their life history. Understanding the biology and behaviour of mosquitoes, especially *Anopheles*, can help people to be proactive towards malaria control. Furthermore, the knowledge of the life history of mosquitoes will help understand how various control strategies work against mosquitoes and enable one to choose an appropriate control strategy in specific situations.

It was observed during the study that students were not motivated to use ITN because they claimed that mosquitoes bit them even before they went to bed. Only few respondents knew or were able to describe different types of mosquitoes but to the majority, all mosquitoes are the same and hence any mosquito bite can result in malaria. Therefore, they did not really appreciate why sleeping under ITNs can prevent malaria. When people have adequate knowledge on the behaviour and life history of mosquitoes, they will better appreciate the need to sleep under ITNs.

Though some respondents had high knowledge related to malaria, misconceptions and inadequate knowledge on malaria also existed among others. When respondents' knowledge on mosquitoes was tested, majority linked mosquitoes to malaria through biting but when the question was reversed (knowledge on malaria) only about 50% linked malaria to mosquitoes. This indicates that people are aware that mosquito plays a role in the transmission of malaria but also have the perception that apart from mosquitoes, there are other causes of malaria. Some went further by citing severe heat from the sun as another cause of malaria. Similar misconceptions among primary school children in some parts of Ghana were also observed by Ayi *et al *[15]. In a related study, education did not appear to have a significant bearing on knowledge of malaria. In that study, however, respondents with secondary or tertiary education were few (6.4%) [16].

From the results of this study, it be can inferred that high level of knowledge on malaria, especially of the link between mosquitoes and malaria, corresponded to high percentage of ITN ownership. This finding is in agreement with other studies [17]. The level of ITN use, however, is reduced by inadequate knowledge on how ITN really works to prevent malaria and by misconceptions about the causes of malaria. This may be the reason why a lot of people own or previously used ITNs but do not use them anymore. A number of studies have shown uncooperative attitudes towards the implementation of malaria control strategies. For instance, insecticide residual spraying (IRS) of houses, the most important malaria control measure in most countries, has been encountering increasing resistance from the target population because most people have a weak perception of (or see no association between) insecticide spraying and mosquito/malaria control. In Zimbabwe, although the indoor spraying programme has been sustained for over 40 years as the mainstay of malaria control, a significant proportion of the target communities continue to have a poor understanding of why their homes should be sprayed [18,19].

Health education appears to be improving malaria-specific knowledge, which in turn is reportedly having some positive impact on ITN ownership at the household level [9] but for people to maintain the use of ITN, education must involve more than production of posters and brochures, or specific instructions regarding, for example, dosage, duration of treatment, and how to treat and hang a net. There is the need to educate people on the biology and behaviour of the vector and the mechanism of a control strategy. For instance, if people understand that there are many species of mosquitoes but only few can transmit the malaria parasite, then they may not stop using ITN because they had been bitten by mosquitoes in the afternoon. Again, if people are made to understand the principles on which ITN or IRS operates to control malaria, then they may make the necessary adjustment or sacrifice to sustain its use. Most of these control strategies depend on the behavior of local malaria vectors such as degree of anthropophily, endophily, endophagy and sporozoite-positive biting time [20]. When the target population does not have much knowledge on mosquito behavior, they do not appreciate the value of control measures and are likely to stop the application of such measures, especially when there are problems (such as discomfort) associated with implementation of the strategies.

Malaria is one of the oldest infectious diseases that have plagued humans throughout history. It is unfortunate that people still have misconceptions about the cause of malaria. Educating people on malaria is very important if indeed malaria can be counted out. While such education may be formal or informal, formal education presents a better forum for delivery of accurate, adequate, and more technical knowledge, through school programmes and courses. Educational systems in malaria endemic countries need to do more to bring positive change in human behaviour that may contribute to transmission of malaria either by promoting the breeding of anopheline mosquitoes or by enhancing man-vector contact.

The curriculum of some subjects was reviewed to assess the contribution it could make to the education of students on malaria. Integrated Science for high school and Science for teachers training college were the subjects reviewed. Integrated science is a compulsory course for all high school students that introduce students to basic sciences. The review of science courses revealed that students in Ghana are exposed to the life cycle of mosquitoes in junior high school, with emphasis on the life cycle and control of mosquitoes. This is encouraging but it falls short of describing the role of mosquitoes in the spread of malaria. 'Infections and Diseases' (Health and Disease, in teacher training college) is another topic through which students in the senior high school could have acquired knowledge on malaria but priority is rather given to other infectious diseases, such as HIV/AIDS, Buruli ulcer and avian flu. Coverage of malaria is apparently left to the discretion of the teachers; hence it is possible for students, especially those who do not take elective science courses, to pass through the educational system up to the university without getting formal knowledge on malaria. This may explain why some senior high and tertiary students still have misconceptions on the cause of malaria. Again this may also be the reason why almost all the parameters measured during the studies were similar in both senior high schools and tertiary institutions. As far as education on malaria is concerned the formal system makes no provision beyond senior high school.

To effect change in behaviour requires sustained long-term activities and not just a few education projects which are mostly done before and after the launch of a malaria programme, and which mostly stop at raising awareness.

There was a significant incidence of ITN usage in the nurses training colleges and it can be due to their programme of study, which exposes them to theoretical and practical aspects of malaria. Ayi *et al *[15] observed that after malaria education which included biology and behaviour of mosquitoes, the misperception that malaria has multiple causes was significantly improved, both among school children and community adults and that the community adults who treated a bed net with insecticide in six months, doubled. The educational system in Ghana can do more in this area especially through the introduction of courses that can expose students to all facets of malaria. A lot of studies have shown that behaviour and social change are critical in malaria control and most countries that have been able to eradicate malaria did so because of these changes. De Zulueta [21] stated that the eradication of malaria from Europe and North America was much more due to changed social conditions than the use of insecticides. This change is only possible through systematic and well-planned education for the people. The formal educational system presents a good opportunity for doing this, as most students are at an age at which behaviour is easier to influence.

## Conclusions

Knowledge and awareness of malaria and ITN were high among all the respondents in the senior high schools and the tertiary institutions; this knowledge, however, was not enough for them to appreciate the use of ITN. Hence, some of them owned ITNs but they did not use them and this may explain why ITN ownership has increased without a corresponding reduction of malaria cases in Ghana. The study again reveals that students did not have adequate knowledge on the biology and behaviour of mosquitoes and of the link between the use of ITN and malaria control. The opinion is that if people will really accept and use ITN or other mosquito control interventions, then creating awareness is not enough but people should be educated on the life history of mosquitoes and the mechanism of the control strategies. This can be achieved through formal education; however, the current involvement of formal education in malaria control in Ghana is not enough. There is existence of programmes or courses at all levels of the education system, that can expose people to all facets of malaria and mosquitoes, but other infectious diseases have been given the priority. If malaria can be counted out, the educational system has a critical role to play, by giving malaria education priority at all levels. In addition, the Ghana Education Service and the Malaria Control Programme can work closely together, especially on malaria education for behaviour change; their combined resources could thus be used to address key issues in a sustained way and key messages could be addressed in a generic manner to avoid confusing the public.

## Competing interests

The authors declare that they have no competing interests.

## Authors' contributions

AAK participated in the design and coordination of the study, carried out the field work and data collection, performed statistical analysis of the data collected and drafted the manuscript. BAM participated in the design of the study and contributed to the writing of the manuscript. All authors read and approved the final manuscript.
